# *Streptomyces rochei* D74 improves tobacco growth and quality by regulating the rhizosphere microecological community

**DOI:** 10.3389/fpls.2026.1748408

**Published:** 2026-03-05

**Authors:** Lumin Zhang, Shiyu Wang, Jiaxin Liu, Yongxian Xu, Yingnan Li, Kun Huang, Hangxian Lai, Junxiang Pu, Xiaoyu Geng, Zhixin Yang, Qiao Guo, Shuanglü Shan

**Affiliations:** 1 Honghe Branch of Yunnan Tobacco Company, Mile, Yunnan, China; 2 College of Natural Resources and Environment, Northwest Agriculture and Forestry (A&F) University, Yangling, Shaanxi, China

**Keywords:** application method, plant growth promotion, rhizosphere microecological regulation, soil nutrient availability, *Streptomyces rochei*, tobacco leaf quality

## Abstract

**Introduction:**

*Streptomyces rochei* D74 promotes growth and enhances quality in crops such as wheat and tomato. However, its potential role and optimal application method in tobacco production remain unclear. This study for the first time investigated the effects of *S. rochei* D74 with different application methods on tobacco growth and quality, soil physicochemical properties, and rhizosphere microbial community structure.

**Methods:**

*S. rochei* D74 was applied via basal application (BA), foliar spray (FS), and their combination (BA-FS) under field conditions. Tobacco growth parameters, leaf yield and quality indicators, soil physicochemical properties, and rhizosphere microbial community structure were analyzed and compared across treatments.

**Results:**

Different microbial treatments promoted tobacco growth compared to the control, as exemplified by notable increases in plant height (by 5.3~10.5%) and stem girth (by 7.0~15.6%), while also reducing the proportion of low-grade leaves (by 10.2~28.4%, *p* < 0.05). Particularly, the BA-FS treatment achieved the highest leaf yield and output value, alongside elevating the contents of total nitrogen (by 29.0~36.2%) and total alkaloids (by 34.3~66.8%) in C3F and B2F grade leaves, increasing the potassium-to-chlorine ratio, and reducing carbohydrate accumulation (e.g., starch). There were corresponding improvements in soil available nutrient contents, including nitrogen, manganese, phosphorus, and iron. Microbial treatments resulted in a lower relative abundance of *Fusarium* in the fungal community, despite not causing a significant shift in bacterial α-diversity. Microbial treatments increased the proportion of positive correlations in bacterial networks and heightened the complexity of fungal networks, thereby likely fostering more cooperative microbial interactions that supported improved nutrient acquisition and plant growth. Mantel analysis revealed that fungal and bacterial community abundances strongly influenced soil nutrient contents and tobacco leaf quality.

**Discussion:**

The findings indicate that combined root and foliar application of *S. rochei* D74 optimally improves tobacco growth and quality by modifying microecological conditions in rhizosphere soil.

## 1 Introduction

Tobacco (*Nicotiana tabacum* L.) is a cash crop that plays a vital role in economic growth and development. China is the largest tobacco producer in the world, consistently leading in both crop area and yield (Teng et al., 2020). Yunnan Province, located in southern China, provides the world’s largest producing area of high-quality flue-cured tobacco and contributes ~45% to the total tobacco production of the country (Sun et al., 2025). High market demand for tobacco drives extensive continuous cropping, and the depletion of land resources exacerbates soil fatigue. Additionally, excessive application of chemical fertilizers and pesticides with insufficient organic amendments accelerates soil degradation, which hinders tobacco growth and impairs crop yield and quality (Huan et al., 2024). Among these issues, the deterioration of soil health, marked by nutrient imbalance and pathogen accumulation, now constitutes the most prominent constraint in tobacco cultivation and, consequently, a primary barrier to the industry’s sustainable development.

Harnessing functional microorganisms for soil amelioration and plant growth promotion represents an innovative approach in agricultural production. In addition to reducing the use of chemical fertilizers, microbial application offers environmental benefits and high efficiency, which aligns with the idea of green development. Functional microorganisms act as pivotal drivers of soil nutrient cycling and plant health. They are able to modify complex rhizosphere microbial networks, thereby bolstering plant resistance and promoting robust growth (Copeland et al., 2025). The genus *Streptomyces* is a representative group of actinomycetes with high stress resistance and capable of producing antibiotics and phytohormones. *Streptomyces* species have been widely applied in agroecosystems to enhance plant tolerance to stress and promote plant growth (Vurukonda et al., 2018). *Streptomyces* species demonstrate practical efficacy in disease management for solanaceous crops. *Streptomyces albidoflavus* serves as an effective bio-fertilizer against potato scab, showing excellent biocontrol potential (Al-Tammar and Khalifa, 2023); likewise, a *Streptomyces*-based spore formulation has exhibited promising control of tomato *Fusarium* wilt (Kirubakaran et al., 2024). In particular, *Streptomyces rochei* performs multiple functions in plant disease control and growth promotion. Secondary metabolites from *S. rochei* exhibit various bioactivities, including antagonism against plant pathogens, promotion of plant growth, and enhancement of plant stress tolerance. The application of *S. rochei* provides a range of growth benefits for crops such as tomato (Luo et al., 2012), wheat (Shan et al., 2025) and apple (Zhang et al., 2016). However, the potential effects of *S. rochei* on plant growth of tobacco remain unclear.

The application of *S. rochei* by different methods considerably influences plant performance. For instance, root drench with *S. rochei* HM85 culture broth improved the growth of sugar beet, as indicated by elevated levels of plant height, fresh plant weight, root length, root diameter, and fresh root weight (Yu et al., 2024). Soil application of *S. rochei* D74 spore powder increased the diversity of rhizosphere bacterial and fungal communities in greenhouse strawberry, thereby promoting plant growth and enhancing fruit yield and quality (Zhou et al., 2025). Moreover, Petri dish assay and field experiment showed that the addition of cell-free culture supernatants from *S. rochei* S32 boosted plant growth of wheat and tomato by modifying rhizosphere microbial community structure. This effect was linked to nitrogen (N) fixation and production of phytohormones, extracellular hydrolases, antibiotics, and siderophores by *S. rochei* S32 based on genomic and metabolomic profiling (Wei et al., 2024). In the context of disease suppression, seed treatment with a spore suspension of *S. rochei* ACTA1551 significantly enhanced tomato seedling resistance against *Fusarium* wilt, while soil incorporation of its culture effectively suppressed disease incidence in greenhouse trials (Kanini et al., 2013). Despite these promising results, no study has systematically compared the efficacy of different *S. rochei* application methods. Identifying the optimal application method of *S. rochei* is crucial for maximizing its benefits to crop production.

This study was conducted in a major tobacco-growing region of Yunnan Province and employed *Streptomyces rochei* D74, a strain with documented plant growth-promoting effects across multiple crops (He et al., 2015; Xi et al., 2022). Its primary methodological novelty lies in implementing and evaluating a novel combined basal application and foliar spray (BA-FS) application strategy at the field scale, and in integrating microbiome co-occurrence network analysis to decipher underlying microbial ecological mechanisms. The aims of this study were to (1) evaluate the effects of *S. rochei* D74 with different application methods—basal application, foliar spray, and their combination—on tobacco growth, yield, and quality; (2) explore the changes in soil physicochemical properties mediated by *S. rochei* D74; and (3) reveal the responses of rhizosphere microbial community structure to *S. rochei* D74. The results of this study broaden the application of the test strain and provide an economical solution for green tobacco production, which, as a directly applicable microbial inoculation technique, serves to alleviate continuous cropping obstacles and enhance nutrient utilization efficiency in the field.

## 2 Materials and methods

### 2.1 Experimental site and cultivation conditions

The experimental site is situated in Houshanhuahai (23°57′ N, 103°35′ E), Gucheng Village, Yangjie Township, Kaiyuan City, Yunnan Province. The study area has an average altitude of approximately 1279 m, characterized by gently sloping terrain and red soil of a light loam texture. The contiguous planting area covers approximately 66.67 ha. The preceding crop was tobacco (variety: Yunyan 87). The fertilization regime (per ha) included 15000 kg of farmyard manure, 225 kg of tobacco-specific compound fertilizer (N:P:K = 12/8/25; Honghe Henglin Chemical Co., Ltd.), 300 kg of calcium fertilizer, 150~225 kg of K_2_SO_4_, and a topdressing of 150 kg of potassium fertilizer applied at the appropriate growth stage. The basic physicochemical properties of the soil are provided in **Supplementary Table 1**.

### 2.2 Source and preparation of experimental microbial inoculants

*Streptomyces rochei* D74 (accession number: KJ145878) were provided by the Resource Biology Laboratory, College of Natural Resources and Environment, Northwest A&F University (Yangling, Shaanxi Province, China). To prepare the field inoculum of *S. rochei* D74, seed culture was initiated by inoculating spores into GN1 medium and incubating for 48 h (Wang et al., 2025). Seed culture expansion (2~5%, v/v) was conducted for 24 h in a primary fermenter containing modified GN1 medium. After subculturing in modified GN1 medium for 48 h, spores were mixed with a bio-organic material (composed primarily of cornmeal substrate, ~30% moisture content) for 2~3 days and then milled to produce powdered solid inoculum (2 × 10^9^ CFU/g). Liquid metabolite extract was prepared by broth fermentation of *S. rochei* D74 (10%, v/v) in modified GN1 medium for 7 days and subsequent supernatant collection via centrifugation.

### 2.3 Experimental design and field treatment arrangement

The experiment was initiated in February 2024, adopting a completely randomized block design. Four treatments were established: control, basal application (BA), foliar spray (FS), and basal application combined with foliar spray (BA-FS) of *S. rochei* D74. Each treatment was replicated three times, resulting in a total of 12 plots. Each plot measured 216 m² (12 m × 18 m), corresponding to approximately 0.067 ha per treatment and containing 1000 tobacco plants.

In the BA treatment, *S. rochei* D74 spore powder (15 kg ha^–1^) was mixed with organic fertilizer and applied at the time of tobacco transplanting. The organic fertilizer, derived from decomposed sheep manure, contained organic matter (65%, *w/w*) and humic acid (15%, *w/w*), with a carbon-to-nitrogen (C/N) ratio of 20:1. The powdered solid inoculant was blended with the organic fertilizer at a ratio of 200:1 (*w/w*), with a viable count of ≥1 × 10^6^ CFU/g, and the mixture was incorporated into the soil prior to tobacco transplanting (Zhou et al., 2025). In non-BA treatments, the same amount of organic fertilizer without the microbial inoculant was applied using the same method.

In the FS treatment, *S. rochei* D74 liquid metabolite extract (diluted 150~200 times, 4500~6000 mL ha^–1^) was evenly sprayed twice—at the root elongation and maturity stages of tobacco (Faria et al., 2023). In non-FS treatments, the same volume of culture medium without microbial inoculation was applied in the same manner.

The BA-FS treatment combined full-strength basal application (at transplanting) with two foliar sprays (at root elongation and maturity), whereas the control treatment followed the abovementioned protocol for the non-BA and non-FS treatments.

The experimental tobacco cultivar was Yunyan 87, sourced directly from the Yunnan Tobacco Administration. The seedlings were transplanted to the field on April 15, 2024, after the seedling stage. A planting configuration with a row spacing of 1.2 m and a plant spacing of 0.6 m was adopted. All other agronomic management practices and plant protection measures followed conventional procedures.

### 2.4 Measurement of tobacco agronomic yield and leaf chemistry

Sampling was conducted during the vigorous growth stage of tobacco in July 2024, with representative plant photographs from each treatment provided in **Supplementary Figure 1**. For each treatment with three replicate plots, two healthy plants per plot were randomly selected and destructively sampled by uprooting the entire plant with its surrounding soil; thus, six biological replicates were obtained per treatment and numbered sequentially in the order of sampling. The agronomic traits of fresh tobacco plants are critical determinants of final dry leaf yield and quality. Therefore, plant height, stem girth, internode distance, maximum leaf length, maximum leaf width and effective leaf number were assessed via field measurement method, while shoot fresh weight and root dry weight were quantified by direct weighing method (Maleki et al., 2011). Root morphology was analyzed using a standard method with an A4 root scanner (Microtek 1800XLplus) and WinRHIZO 3.0 image analysis software (Regent Instruments Inc.), focusing on key parameters such as total root length, total root surface area, average root diameter and total root volume (Himmelbauer et al., 2004).

According to the National Standard GB 2635-1992, flue-cured tobacco is classified into 42 grades based on seven key appearance factors, including maturity, leaf structure, identity, oil content, color, length, and residual injury. These tiers include premium-grade (e.g., C3F, B2F), medium-grade (e.g., C3L, B2L), and low-grade tobacco (e.g., B4L, X3L, B2K). During the mature harvest period of tobacco leaves, the tobacco leaves of each treatment were harvested and cured separately, graded according to the standard, the proportion of tobacco leaves at all levels was calculated, the total weight of tobacco leaves at all levels was weighed, and the total yield per ha was calculated. Then the output value per ha was calculated according to the purchase price of different grades of tobacco leaves in the same year (Tang et al., 2020).

As representative benchmarks of flue-cured tobacco, the premium-grades C3F (mid-position, third-grade, orange-yellow, flexible) and B2F (upper-position, second-grade, orange-yellow, flexible) derive their core market value from their chemical composition, which directly governs sensory quality, combustion characteristics, and industrial usability (Zhou et al., 2025). The chemical components of tobacco leaves were selected 3.0 kg of C3F and B2F grade tobacco samples for detection and analysis. The contents of water-soluble total sugars and reducing sugars were determined by segmented flow analysis using the anthrone colorimetric method (Hosfield et al., 1982). The contents of total alkaloids, total nitrogen (N), chloride (Cl^−^), and potassium (K^+^) in tobacco leaves were quantitatively determined using UV spectrophotometry, the Kjeldahl method, silver nitrate titration, and flame photometry, respectively (Johnstone and Plimmer, 1959).

### 2.5 Analysis of soil physicochemical traits and rhizosphere microbial sequencing

The aforementioned 24 tobacco samples (four treatments with six replicates each) were carefully preserved and transported to the laboratory for further processing. From each plant, two types of soil samples—root-zone soil and rhizosphere soil—were collected. Root-zone soil was obtained by gently shaking the excavated root system to collect the loosely adhered soil. Approximately 500 g of this soil was air-dried, ground, and passed through a 20-mesh sieve for subsequent analysis of soil nutrients and trace elements. Rhizosphere soil, defined as the fraction tightly adhering to the root surface, was removed with a sterile brush, placed into sterile centrifuge tubes, and stored at -80 °C for soil microbial community analysis.

Soil pH was measured using a UB-7 pH meter (Sartorius, Germany) at a water-to-soil ratio of 1:2.5 (*w*/*v*) (Li et al., 2018). Soil organic matter content was determined by the potassium dichromate oxidation method (Fomenko and Kaziuta, 2023). Hydrolyzable N was measured by the alkaline hydrolysis diffusion method according to the Chinese forestry standard LY/T 1229-1999. Available phosphorus (P) was extracted with NaHCO_3_ solution and quantified by the vanadium molybdate yellow colorimetric method (Bhatia et al., 2021). Available K was extracted with ammonium acetate and determined by flame photometry. Exchangeable cations, including available zinc (Zn), manganese (Mn), iron (Fe), copper (Cu), and exchangeable Ca, were displaced with ammonium acetate and detected by atomic absorption spectrometry (Alekseeva et al., 2011).

Rhizosphere soil samples collected during the tobacco vigorous growth stage were sent to Shanghai Biozeron Biotechnology Co., Ltd. for 16S and ITS rRNA gene amplicon sequencing. DNA extraction was performed using the FastDNA Spin Kit for Soil (MP Biomedicals, Santa Ana, CA, USA), followed by quantification and integrity assessment. The hypervariable regions of the 16S rRNA gene were amplified with the primers 341F (5’-CCTAYGGGRBGCASCAG-3’) and 806R (5’-GGACTACNNGGGTATCTAAT-3’). Similarly, the ITS region was amplified using the fungal-specific primers ITS1F (5’-CTTGGTCATTTAGAGGAAGTAA-3’) and ITS2R (5’-GCTGCGTTCTTCATCGATGC-3’). Following PCR amplification, the products were purified, and the library was checked for fragment size distribution and concentration. Libraries that passed quality control were sequenced on an Illumina NovaSeq platform (USA) using a PE250 strategy. Raw sequencing data were processed using readfq (v1.0) to generate high-quality Clean Data (He et al., 2013). Denoising was performed with the DADA2 algorithm within QIIME2 (v2024.10) to obtain Amplicon Sequence Variants (ASVs), which represent sequences with 100% similarity (Bokulich et al., 2018). Following the construction of the ASV feature table, representative sequences of ASVs were taxonomically annotated using the RDP classifier (v2.2) against reference databases with a confidence threshold of 0.6. Specifically, the Silva (v13.8) and UNITE (v8.2) databases were employed for the annotation of 16S bacterial and ITS fungal sequences, respectively (Wang et al., 2007).

### 2.6 Data processing statistical analysis and bioinformatics methods

From the six biological replicates, five were randomly selected for use in all subsequent analyses. Data analysis and visualization were performed using Excel 2010 for bar chart construction and SPSS 24.0 for statistical analysis. Data were analyzed by one-way ANOVA. Following a significant overall effect (*p* < 0.05), means were compared using Tukey’s HSD *post hoc* test. Results are presented as mean ± SE. We employed a fuzzy comprehensive evaluation method to assess different microbial application methods. The final composite index was derived by normalizing key soil and leaf indicators (**Supplementary Table 2**) into membership scores and weighting them based on their average absolute correlation coefficients (**Supplementary Table 3**).

To ensure the comparability of results, all analyses including alpha and beta diversity, taxonomic composition, differential abundance, and correlation analyses were performed on a rarefied feature table generated by the q2−feature−table plugin in QIIME 2, with rarefaction depth set to the minimum sequence count across all samples to mitigate biases from uneven sequencing effort; a rarefaction curve provided in the **Supplementary Figure 3** confirms that the sequencing depth was sufficient for downstream analyses. Microbial α-diversity was analyzed using the Wilcoxon rank-sum test in R software (v3.4.1). β-Diversity was assessed via principal coordinate analysis (PCoA) based on unweighted UniFrac distance matrices, and the community structure differences were tested using Adonis. Furthermore, bar charts were generated to depict the average relative abundances of the top 15 taxa at the genus level.

The construction of microbial correlation network was based on the bacterial and fungal species annotation table, and the species that appeared in more than 50% of all samples were retained. The Hmisc package in R software was used to analyze the correlation network of the microbial community at the species level, and the Pearson correlation coefficient was calculated. The correlation (*r*) and significance (*p*) matrices were generated, and the species data with |*r*| > 0.80 and *p* < 0.05 were imported into the software Gephi (v0.9.7) to draw the correlation network and calculate its topological properties. The relationship between microbial communities and environmental factors (soil and plant) was examined using Mantel tests implemented with the mantel function in the vegan package for R. This test correlated the Bray-Curtis dissimilarity matrix of the microbial community with the matrix of environmental factors using Spearman’s rank correlation. The results were visualized using the ggplot2 and corrplot packages.

## 3 Results

### 3.1 Root and foliar application of *S. rochei* D74 additively optimizes tobacco yield and economic value

**Figure 1:**
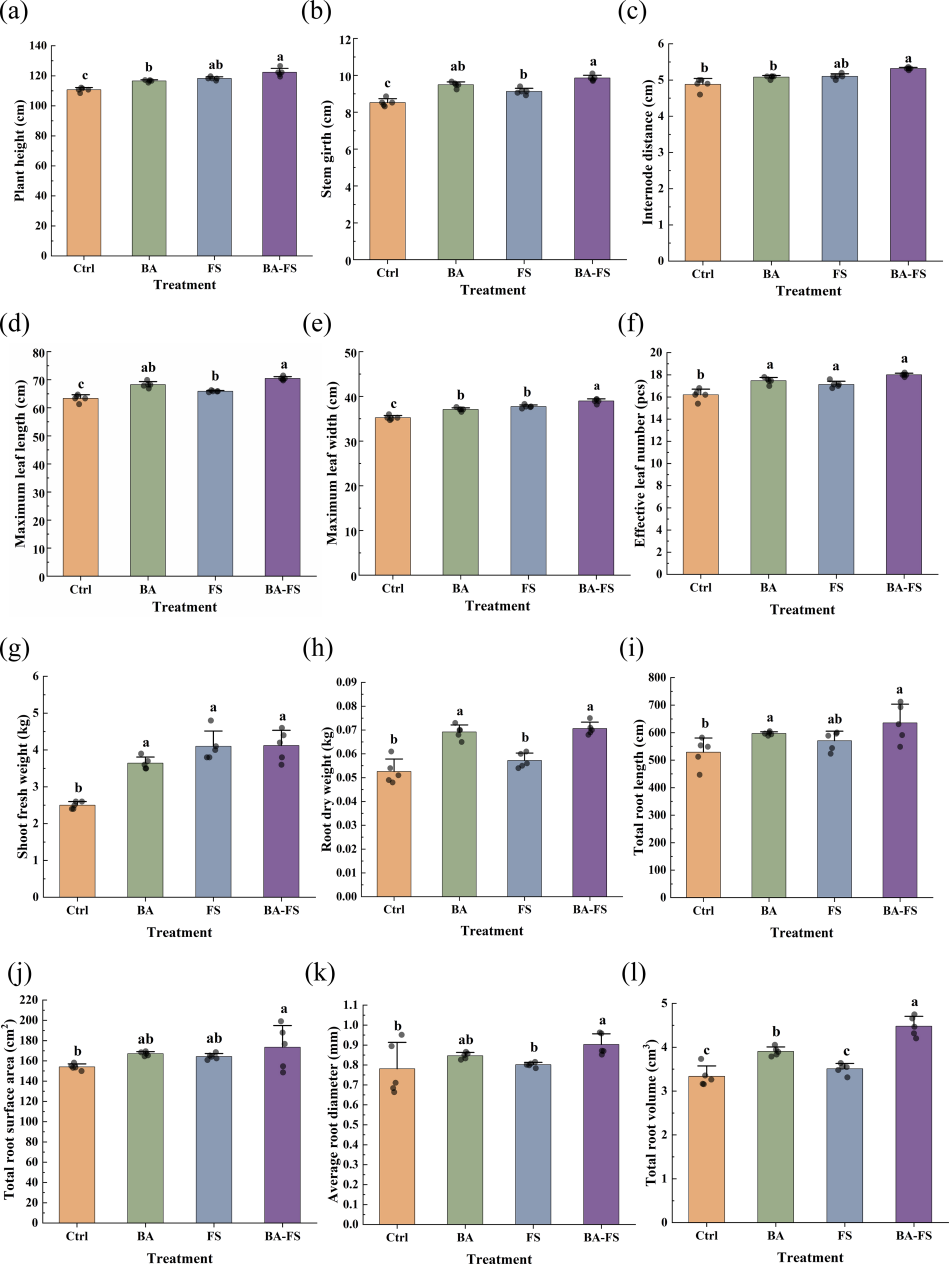
Effects of different microbial application methods on agronomic traits of tobacco. **(a)** Plant height. **(b)** Stem girth. **(c)** Internode distance. **(d)** Maximum leaf length. **(e)** Maximum leaf width. **(f)** Effective leaf number. **(g)** Shoot fresh weight. **(h)** Root dry weight. **(i)** Total root length. **(j)** Total root surface area. **(k)** Average root diameter. **(l)** Total root volume. Ctrl = Sterile organic fertilizer (root) + sterile medium (foliar spray); BA = basal application; FS = foliar spray; BA-FS = basal application combined with foliar spray. Error bars represent standard deviation of the mean (*n* = 5). Different letters above the error bars indicate significant differences among treatments (one-way ANOVA with Tukey’s HSD test, *p* < 0.05).

Irrespective of the method used, microbial application significantly promoted stem growth of tobacco plants (**Figures 1a–c**). Compared to the control, higher plant height was observed with BA (by 10.5%), FS (by 6.8%), and BA-FS (by 5.3%). Stem girth also increased with BA (by 15.6%), FS (by 7.0%), and BA-FS (by 11.3%). Leaf growth positively responded to various microbial treatments (**Figures 1d–f**). In particular, BA-FS resulted in the greatest increase of ~11% in maximum leaf length, maximum leaf width, and effective leaf number. Consistently, plant biomass accumulated to higher levels under microbial treatments, with shoot fresh weight rising by 32.9~51.8% (**Figures 1g, h**). The total length, total surface area, average diameter, and total volume of belowground roots generally improved in microbial treatments, albeit not always significantly (**Figures 1i–l**). The results indicate that the application of *S. rochei* D74 boosted the above- and belowground growth of tobacco plants. Combined root and foliar application was superior to single applications in improving the agronomic traits of tobacco.

**Figure 2:**
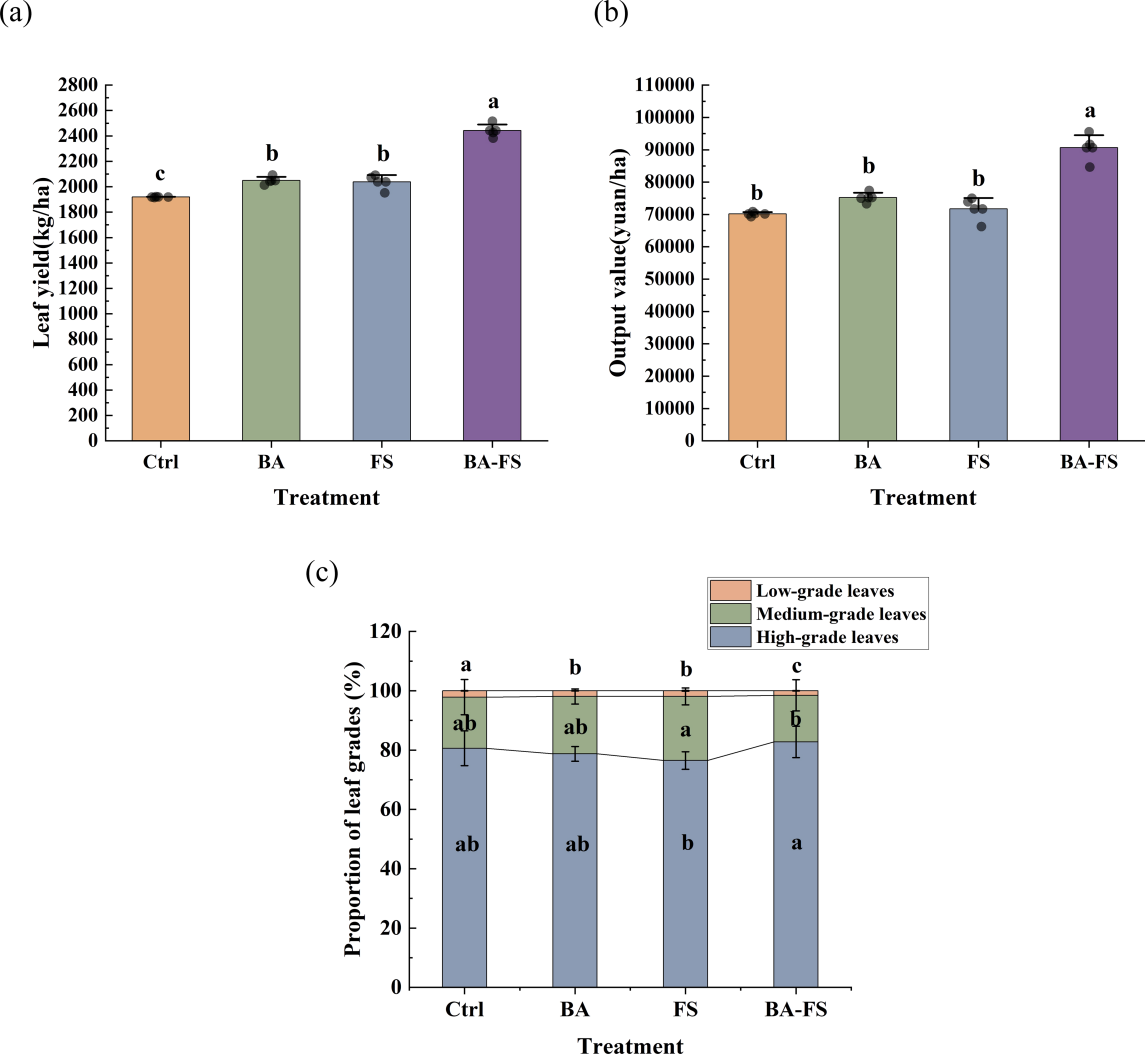
Responses of flue-cured tobacco yield and grade to microbial application using different methods. **(a)** Leaf yield. **(b)** Output value. **(c)** Proportion of leaf grades. Error bars represent standard deviation of the mean (*n* = 5). Different letters above the error bars indicate significant differences among treatments (one-way ANOVA with Tukey’s HSD test, *p* < 0.05).

Greater leaf yield and output value of flue-cured tobacco were obtained under microbial treatments compared to the control, with BA-FS achieving the maximum increase of 27.2% and 29.2%, respectively (**Figures 2a, b**). BA-FS resulted in a 2.1% higher proportion of high-grade leaves than the control, while BA and FS respectively elevated the proportion of medium-grade leaves by 2.10% and 4.35%. The total proportion of high- and medium-grade leaves increased by 0.23%~0.61% under microbial treatments, accompanied by 10.2~28.4% decrease in the proportion of low-grade leaves (**Figure 2c**). The results indicated that, relative to other methods, the combined root and foliar application of *S. rochei* D74 led to a superior improvement in the yield and grade of flue-cured tobacco.

### 3.2 Additive response of *S. rochei* D74 root and foliar application enhances leaf chemistry in high-grade tobacco

The chemical quality of high-grade tobacco leaves showed distinct changes across treatments (**Supplementary Figure 4**). The accumulation of total N and alkaloids in C3F and B2F grade leaves increased with BA and BA-FS compared to the control, whereas the total alkaloid accumulation in B2F grade leaves decreased with FS. For example, BA resulted in higher total alkaloid content in C3F and B2F grade leaves by 66.8% and 38.5%, respectively, along with ~34.6% greater total N content. BA-FS elevated the total alkaloid content in C3F and B2F grade leaves by 40.6% and 34.3%, respectively, together with ~31.0% increase in their total N content (**Supplementary Figures 4a, b, g, h**).

Compared to the control, the accumulation of carbohydrates (total sugars, reducing sugars, starch) in C3F grade leaves was reduced under microbial treatments. Similarly, B2F grade leaves showed lower carbohydrate accumulation with BA and BA-FS, despite their slightly higher contents of total sugars and reducing sugars with FS (**Supplementary Figures 4c, d, i, j**). Various microbial treatments also improved the K^+^ content in C3F grade leaves (by 16.8~21.8%) and B2F grade leaves (by 24.6~34.2%), resulting in notably higher K/Cl ratios (**Supplementary Figures 4e, k, m**). In short, basal application of *S. rochei* D74, alone and in combination with foliar spray, improved the chemical quality of high-grade leaves, contributing to high-quality tobacco production.

### 3.3 *S. rochei* D74 combined root and foliar application additively boosts soil nutrient supply for improved tobacco growth

Soil pH (7.70~7.85) did not significantly change among various treatments (**Table 1**). Compared to the control, soil available Cu content was 67.9~97.5% higher under microbial treatments. Similarly, the content of exchangeable Ca increased by 10.4~21.3%. Soil hydrolyzable N and available Mn contents showed comparable increase of 11.7~18.2% with BA and BA-FS. Additionally, BA-FS increased soil available P and available Fe contents by 63.4% and 13.9%, respectively. The results imply that combined root and foliar application of *S. rochei* D74 created favorable soil conditions for tobacco growth by improving the supply of key nutrient elements.

**Table 1:** Soil physicochemical properties affected by different microbial application methods.

Treatment	pH value	Organic matter (g/kg)	Hydrolyzable N (mg/kg)	Available P (mg/kg)	Available K (mg/kg)
Ctrl	7.73 ± 0.21 a	16.92 ± 2.73 a	69.20 ± 4.44 b	16.34 ± 6.45 b	500.80 ± 84.66 a
BA	7.79 ± 0.12 a	19.06 ± 0.83 a	80.00 ± 4.64 a	25.66 ± 1.61 ab	415.60 ± 123.68 a
FS	7.78 ± 0.29 a	18.90 ± 2.59 a	70.40 ± 4.51 b	30.80 ± 9.92 ab	438.60 ± 214.15 a
BA-FS	7.82 ± 0.23 a	19.66 ± 0.80 a	**81.80 ± 2.17 a**	**36.98 ± 16.73 a**	423.60 ± 77.09 a
Treatment	Available Zn (mg/kg)	Available Mn (mg/kg)	Available Fe (mg/kg)	Available Cu (mg/kg)	Exchangeable Ca (mg/kg)
Ctrl	1.25 ± 0.05 a	4.80 ± 0.16 b	2.74 ± 0.09 b	0.81 ± 0.23 b	2033.60 ± 40.43 d
BA	1.28 ± 0.08 a	5.36 ± 0.36 a	3.08 ± 0.22 a	1.36 ± 0.18 a	2381.40 ± 64.76 b
FS	1.26 ± 0.05 a	5.02 ± 0.33 ab	2.92 ± 0.31 ab	1.48 ± 0.25 a	2246.00 ± 53.56 c
BA-FS	1.29 ± 0.06 a	**5.42 ± 0.39 a**	**3.12 ± 0.07 a**	**1.60 ± 0.16 a**	**2467.00 ± 94.81 a**

Ctrl, Sterile organic fertilizer (root) + sterile medium (foliar spray); BA, basal application; FS, foliar spray; BA-FS, basal application combined with foliar spray. Data are presented as the mean ± standard deviation (*n* = 5). For each variable, values followed by different letters in the same column are significantly different among treatments (one-way ANOVA with Tukey’s HSD test, *p* < 0.05). The bold values denote statistically significant increases in the measured parameters (e.g., p < 0.05).

### 3.4 Membership function analysis validates the optimality of combined root-foliar application

To comprehensively evaluate the performance of the different microbial application methods, we employed a membership function analysis (as detailed in the **Supplementary Figure 2**) to calculate a composite score reflecting the conformity of key soil physicochemical properties with tobacco yield and quality indices. The control group obtained a conformity score of 0.442, lower than those of microbial treatments (0.472~0.549). BA, FS, and BA-FS improved the conformity score by 6.8%, 9.7%, and 24.2%, respectively. Among them, BA-FS achieved the highest conformity score, which mirrors the patterns in soil properties and tobacco traits. Taken together, the results confirm that combining basal application with foliar spray was the optimal scheme for application of *S. rochei* D74 in tobacco fields.

### 3.5 *S. rochei* D74 reshapes rhizosphere microbiota to sustain plant health

**Figure 3:**
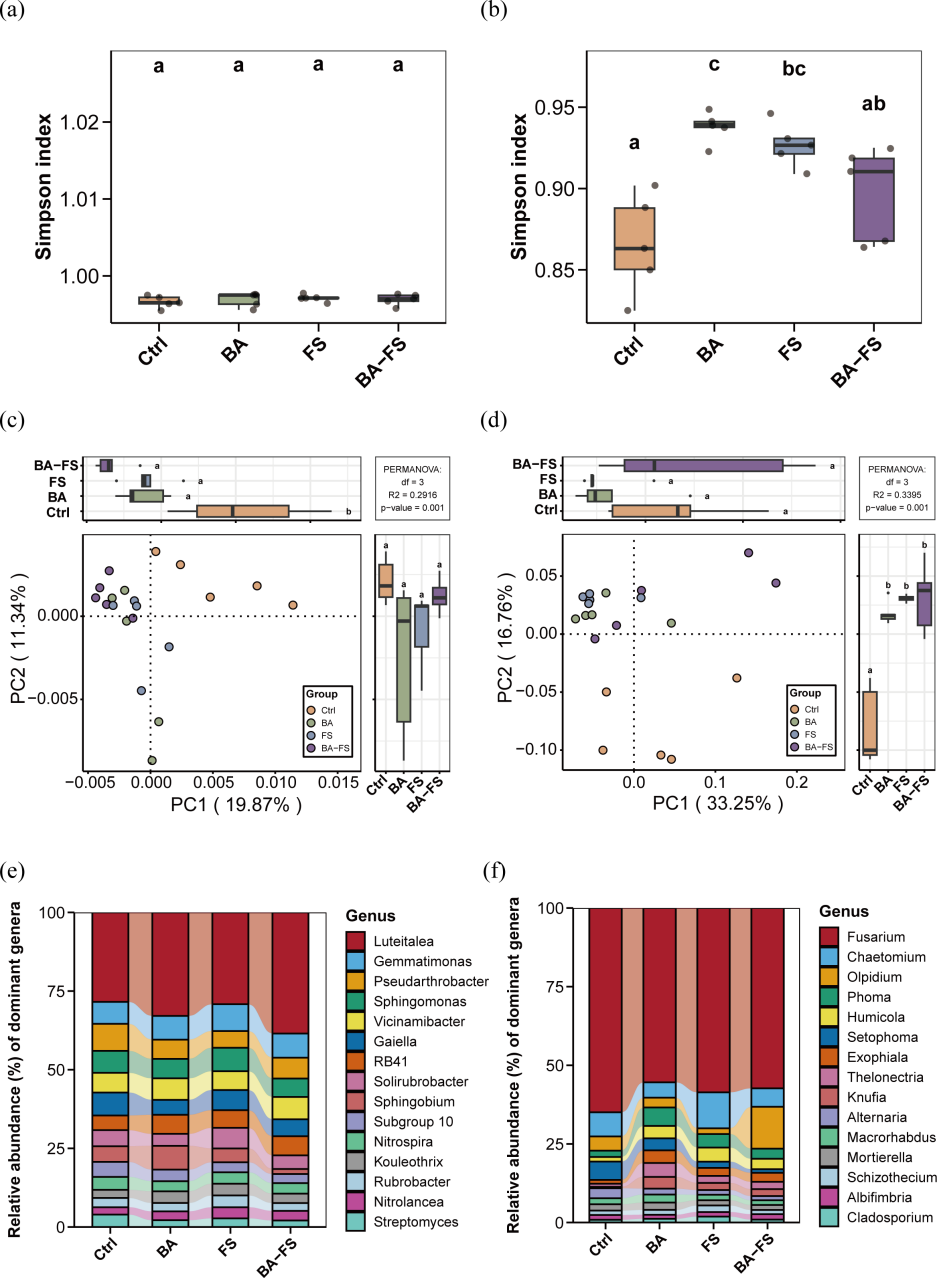
Diversity and abundance patterns of rhizosphere microbial communities under microbial application using different methods. **(a)** Bacterial Simpson index. **(b)** Fungal Simpson index. **(c)** Bacterial PCoA. **(d)** Fungal PCoA. **(e)** Bacterial community composition. **(f)**. Fungal community composition. Ctrl, Sterile organic fertilizer (root) + sterile medium (foliar spray); BA, basal application; FS, foliar spray; BA-FS, basalapplication combined with foliar spray. Different letters indicate significant differences among treatments (one-way ANOVA with Tukey’s HSD test, *p* < 0.05).

To explore whether different application methods affect tobacco plant growth and quality through regulating rhizosphere microbial community structure, we analyzed microbial diversity and community composition in soil samples. The Simpson index was used to assess the α-diversity of bacterial and fungal communities. Compared to the control, bacterial α-diversity showed no significant differences in neither the individual (BA, FS) nor the combined (BA-FS) treatments (**Figure 3a**). Fungal α-diversity trended lower under microbial treatments (as indicated by higher Simpson index), and there was significant difference between BA and FS treatments (**Figure 3b**). The PCoA results highlighted variations in microbial community composition among different treatments (**Figures 3c, d**). Samples of each microbial treatment group were clearly separated from control samples in β-diversity distribution. For bacteria, the first two principal components accounted for 19.87% and 11.34% of total variation, respectively (*p* = 0.001 by Adonis test). For fungi, the first two principal components accounted for 33.25% and 16.76% of total variation, respectively (*p* = 0.001 by Adonis test). The results indicate that the application of *S. rochei* D74 by different methods altered the diversity and composition of rhizosphere microbial communities associated with tobacco.

Across the 20 soil samples (five per treatment), a total of 14,458 bacterial ASVs were obtained and classified into 931 genera, 352 families, 209 orders, 93 classes, and 42 phyla. Additionally, 2,052 fungal ASVs were classified into 484 genera, 215 families, 96 orders, 34 classes, and 7 phyla. To characterize the differentially abundant taxa between the control and microbial treatments, we plotted the top 15 genera in terms of their relative abundance. The bacterial community contained a high proportion of *Luteitalea* (28.4~38.5%), followed by *Gemmatimonas*, *Pseudarthrobacter*, *Sphingomonas*, *Vicinamibacter*, *Gaiella*, *RB41*, *Solirubrobacter*, *Sphingobium*, *Subgroup 10*, *Nitrospira*, *Kouleothrix*, *Rubrobacter*, *Nitrolancea*, and *Streptomyces* (**Figure 3e**). In the fungal community, *Fusarium* accounted for the highest proportion (55.4~65.0%), followed by *Chaetomium*, *Olpidium*, *Phoma*, *Humicola*, *Setophoma*, *Exophiala*, *Thelonectria*, *Knufia*, *Alternaria*, *Macrorhabdus*, *Moritella*, *Schizothecium*, *Albifimbria*, and *Cladosporium* (**Figure 3f**). Several bacterial genera associated with soil nutrient cycling, such as *Luteitalea*, *Gemmatimonas*, and *RB41*, showed higher relative abundances under microbial treatments compared to the control. The fungal genus *Fusarium* decreased in relative abundance by 3.9%~12.2% under microbial treatments, with *Fusarium oxysporum* showing a significant reduction of up to 26.67% under BA-FS treatment (*p* < 0.05; **Supplementary Figure 5**).

### 3.6 *S. rochei* D74 strengthens the stability of rhizosphere microbial networks

**Figure 4:**
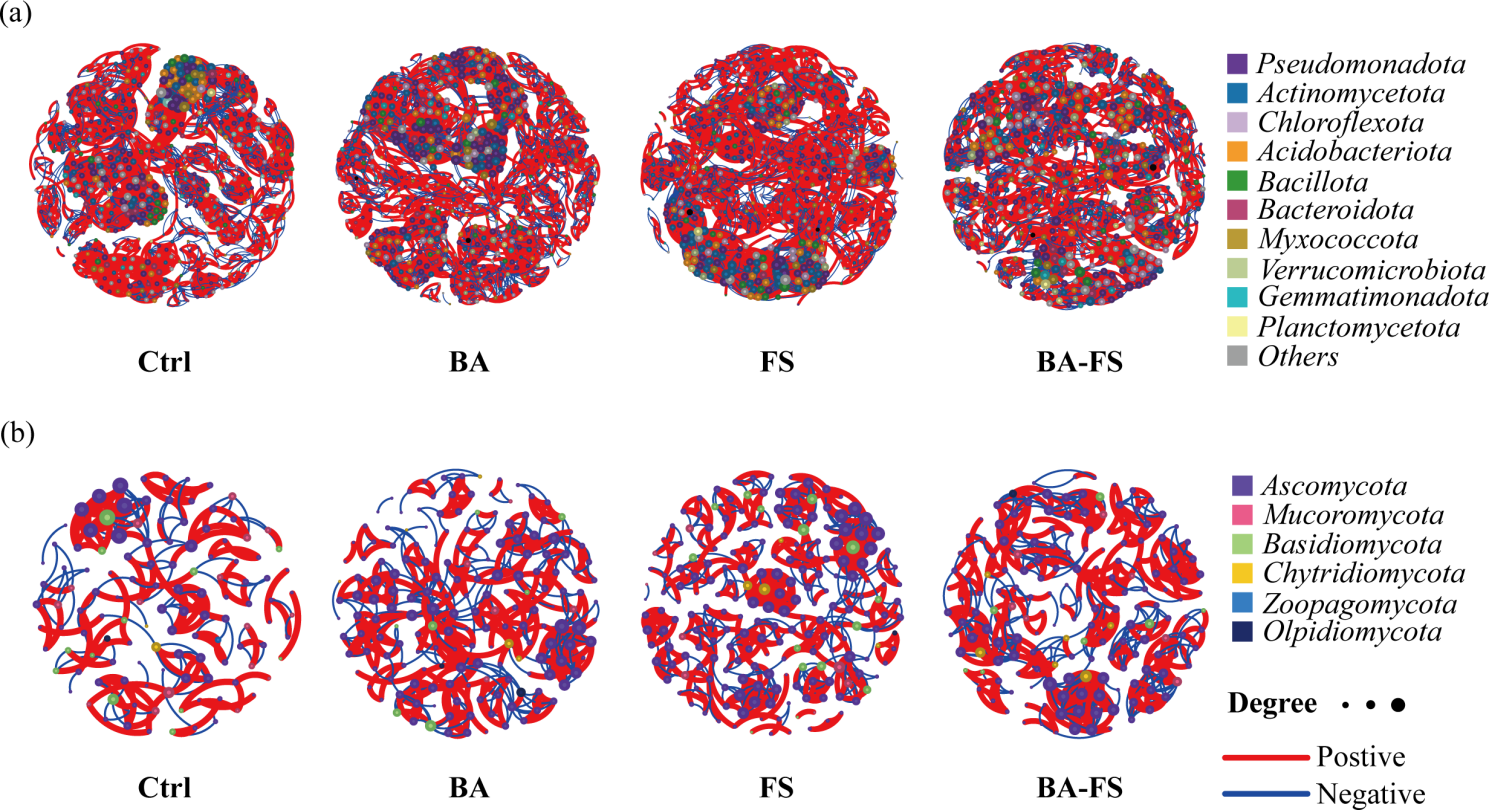
Rhizosphere microbial networks shaped by different microbial application methods. **(a)** Bacterial networks. **(b)** Fungal networks. Only significant correlations are shown (|*r*| > 0.80 and *p* < 0.05). Nodes **(circles)** represent species; the node size is proportional to species relative abundance and the node color corresponds to phylum. Edges **(lines)** represent correlation between two nodes; red lines indicate positive correlations and blue lines indicate negative correlations; the number of lines reflects the connection density. Ctrl, Sterile organic fertilizer (root) + sterile medium (foliar spray); BA, basal application; FS, foliar spray; BA-FS, basal application combined with foliar spray.

Network analysis revealed distinct co-occurrence patterns of rhizosphere microbial communities among treatments (**Figure 4**). Both BA and BA-FS resulted in notably higher eigenvector centrality in the bacterial network compared to the control, implying heightened importance of core microbiota (**Table 2**). When FS was applied, the bacterial network showed slightly higher number of edges and average degree, despite containing fewer nodes. In all cases, the rhizobacterial interaction network exhibited significantly increased robustness, demonstrating enhanced network stability (**Supplementary Figure 6a**). Within this network, the phylum *Pseudomonadota* consistently held a pivotal position and maintained the highest number of connections with other taxa, underscoring its critical ecological function (**Figure 4a**). The fungal networks from various microbial treatments displayed greater numbers of nodes and edges compared to the control (**Figure 4b**), indicating more complex species interactions, which were accompanied by a significant increase in network robustness (**Supplementary Figure 6b**). The modularity of fungal networks was generally improved under microbial treatments, by 3.86-fold with BA and by 1.23-fold with BA-FS.

**Table 2:** Topological properties of rhizosphere microbial networks under microbial application using different methods.

Microbial group	Treatment	Edges (*n*)	Nodes (*n*)	Average degree	Average path length	Modularity	Average clustering coefficient	Eigenvector centrality	Positive correlations (%)	Negative correlations (%)
Bacteria	Ctrl	5335	574	18.589	10.156	8.387	0.774	0.083	52.86	47.14
	BA	4760	573	16.614	8.472	5.342	0.737	0.118	54.54	45.46
	FS	5346	540	19.800	7.402	6.645	0.733	0.087	53.57	46.43
	BA-FS	3387	530	12.781	8.468	8.235	0.749	0.160	53.00	47.00
Fungi	Ctrl	203	123	3.301	1.825	4.574	0.853	0.020	54.68	45.32
	BA	414	177	4.678	3.859	22.254	0.744	0.048	50.72	49.28
	FS	544	211	5.156	3.083	4.878	0.809	0.055	54.96	45.04
	BA-FS	503	176	5.716	3.758	10.203	0.770	0.043	52.09	47.91

Ctrl, Sterile organic fertilizer (root) + sterile medium (foliar spray); BA, basal application; FS, foliar spray; BA-FS, basal application combined with foliarspray..

To decipher the network interaction patterns of rhizosphere microbial communities, we calculated the proportions of positive and negative correlations in each network (**Table 2**). The bacterial networks from microbial treatments contained 54.5% (BA), 53.6% (FS), and 53.0% (BA-FS) of positive correlations, higher than 52.9% of the control. However, in fungal networks, there were lower proportions of positive correlations with BA (50.7%) and BA-FS (52.1%) compared to the control (54.7%). The results suggest that bacterial communities likely established more cooperative associations between species after application of *S. rochei* D74. Basal application alone and in combination with foliar spray potentially stimulated species competition within fungal communities.

### 3.7 Association analysis of rhizosphere microbial community with soil properties and tobacco traits

**Figure 5:**
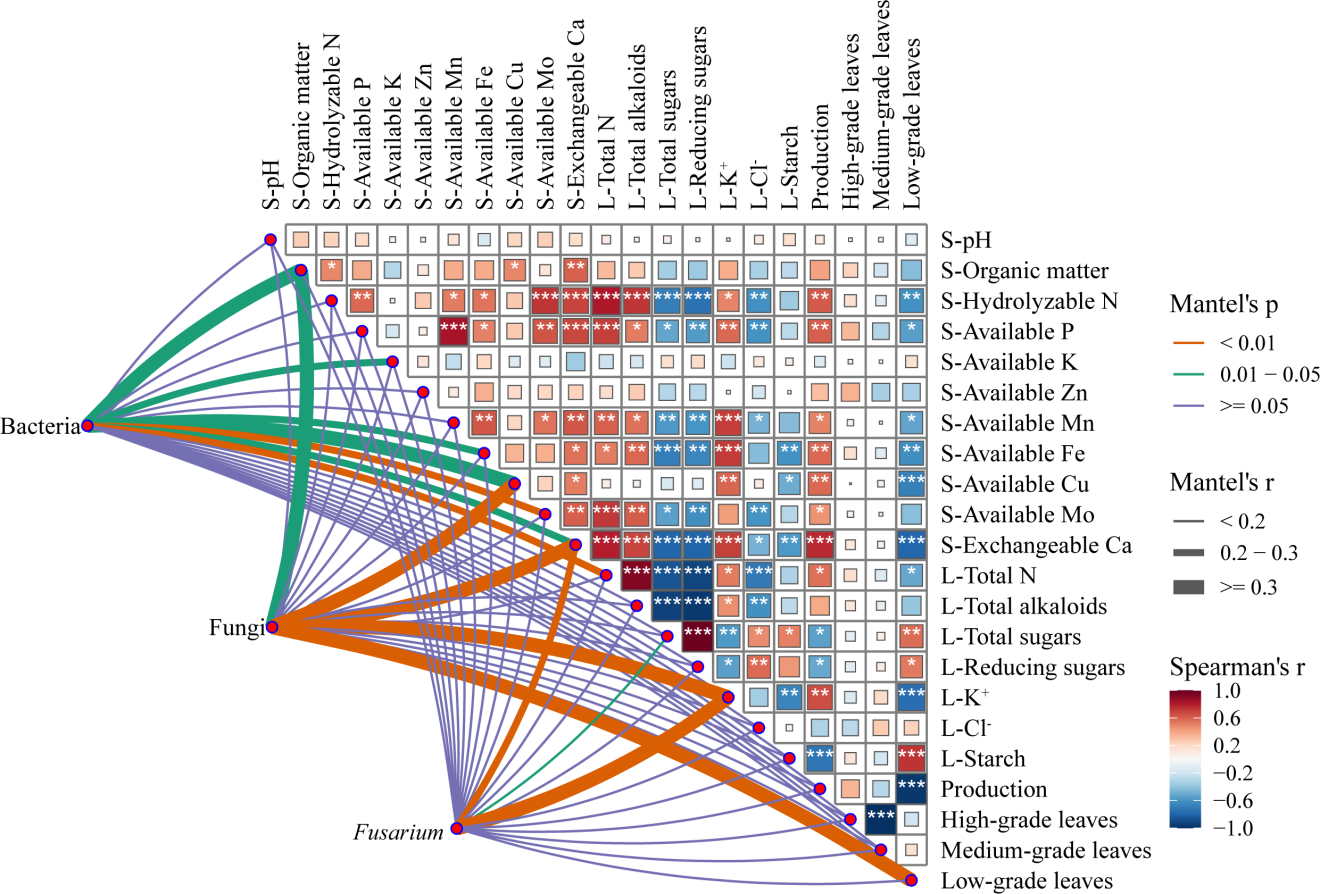
Relationships of rhizosphere microbial communities, soil physicochemical properties, tobacco yield, and leaf quality. The color gradient on the right indicates Spearman’s correlation coefficients between soil and plant factors (* *p* < 0.05, *p* < 0.01, and *** *p* < 0.001). The lines on the left stand for distance-based correlations between microbial abundances and soil and plant factors; the line width corresponds to Mantel’s *r* statistic and the line color indicates statistical significance (*p* value) based on 999 permutations. S-, soil; L-, leaf.

Mantel tests revealed close relationships between soil physicochemical properties, tobacco leaf yield, C3F grade leaf quality, and rhizosphere microbial community structure (**Figure 5**). With respect to soil properties, multiple nutrient contents were strongly correlated with fungal community abundance (*r* > 0.3). Significant correlations also emerged between some nutrient contents and bacterial community abundance, despite relatively low strength. For example, soil available Cu and exchangeable Ca contents were significantly correlated with both bacterial and fungal community abundances (*p* < 0.05).

Regarding tobacco traits, leaf K^+^ content was significantly correlated with fungal community abundance and *Fusarium* abundance (*p* < 0.01). The proportion of low-grade leaves was significantly correlated with fungal community abundance (*p* < 0.01). There was a significant correlation between leaf N content and bacterial community abundance (*p* < 0.01). Moreover, leaf yield was positively correlated with soil exchangeable Ca content (*p* < 0.001) and negatively correlated with leaf starch content (*p* < 0.001). The proportion of low-grade leaves was positively correlated with the contents of leaf starch (*p* < 0.001), total sugars (*p* < 0.05), and reducing sugars (*p* < 0.05). Negative correlations were observed between the proportion of low-grade leaves and the contents of soil available Cu (*p* < 0.001), exchangeable Ca (*p* < 0.001), and leaf K^+^ (*p* < 0.001). These results reveal the complex associations among soil properties, microbial communities, and tobacco plant physiology, providing primarily associative evidence that may inform strategies for sustainable tobacco production.

## 4 Discussion

*Streptomyces* spp. play multifunctional roles in regulating plant growth, systemic resistance, and soil microecology (Liu et al., 2024). Despite *S. rochei* demonstrates growth benefits to various crops (Amaresan et al., 2018), its potential role and optimal application method in tobacco production systems remain unclear. Therefore, we applied *S. rochei* D74 under field conditions using three different methods and systematically evaluated the effects on tobacco growth and rhizosphere microecology. We found that the application of *S. rochei* D74 improved the soil microenvironment and optimized rhizosphere microbial community structure, thereby enhancing tobacco yield and quality. By comparing the different methods, we identified basal application combined with foliar spray (BA-FS) as the optimal scheme for field application of *S. rochei* D74. These findings elucidate the mechanisms underlying the plant growth-promoting effects of *S. rochei* D74 on tobacco and provide a technical strategy for green tobacco production.

### 4.1 Optimal scheme for field application of *S. rochei* D74

Basal application and foliar spray are popular methods for application of functional microorganisms, exhibiting distinct advantages in crop growth improvement (Clemente et al., 2016; Francioli et al., 2025). Our results showed that basal application of the spore powder from *S. rochei* D74 (BA) prominently improved soil physicochemical properties. Foliar spray of the metabolite extract from *S. rochei* D74 (FS) principally enhanced the chemical quality of tobacco leaves. This finding echoes prior studies that used different functional microorganisms and crop species. For instance, Wang and Yang (2024) reported that the rhizosphere microenvironment of blueberry was modified by basal application of a *Pseudomonas* strain, which facilitated plant growth. Huang et al. (2020) showed that basal application of microbial inoculants contributed to rapid nutrient supply and improved physiological traits in peanut plants. Brankov et al. (2020) found that corn growth and yield were improved by foliar application of a microbial-based fertilizer. Compared to single treatments, the BA-FS treatment maximized the potential of *S. rochei* D74 to enhance soil nutrient supply and improve tobacco leaf yield and quality. By integrating basal application (BA) and foliar spray (FS), it achieved integrated management and functional complementarity of both the rhizosphere and phyllosphere environments. The combined treatment also demonstrated the best performance in comprehensive conformity evaluations across different assessment methods, further confirming its overall advantage. In summary, the integrated strategy of combined basal and foliar application provides an effective approach for utilizing *S. rochei* D74 in tobacco production, which facilitates the simultaneous improvement in rhizospheric nutrient supply and phyllospheric physiological activity. However, the optimal application ratio and timing within this strategy require further in-depth study to maximize its practical efficacy.

### 4.2 *S. rochei* D74 enhances tobacco growth and quality by regulating soil properties and plant physiology

Soil nutrient status directly affects plant growth, development, and yield. Long-term continuous cropping of tobacco leads to soil degradation and nutrient imbalance, thus retarding plant growth (Yu et al., 2024). The BA-FS treatment effectively increased soil nutrient contents (e.g., hydrolyzable N, available Fe), indicating accelerated nutrient transformation and modified nutrient composition in soil after combined application of *S. rochei* D74. A previous study has reported that *Streptomyces* inoculants enhance fertilizer use efficiency by activating nutrients and facilitating their transformation processes (Ameh et al., 2024). Treatment with *S. rochei* D74 also enhanced agronomic traits, including plant height, stem girth, internode distance, and leaf length and width in tobacco, resulting in notably higher shoot biomass. The effects of microbial application on plant aboveground growth can be attributed to the optimization of rhizosphere soil properties and enhancement of root metabolic activity (Xi et al., 2025).

Leaf chemical composition serves as the core criterion for evaluating flue-cured tobacco quality. The BA-FS treatment markedly improved the coordination of chemical components in tobacco leaves as exemplified by C3F grade leaves. Specifically, the total alkaloid content of leaf samples increased to 3.4%, falling within the optimal range for high-quality tobacco leaves (1.5~5.0%) (Schmeltz and Hoffmann, 1977). The K/Cl ratio of leaf samples rose to 10.4%, enhancing the ash-holding capacity of tobacco leaves during combustion. The total sugar content of leaf samples decreased to 25.0%, meeting the standard for high-quality tobacco (18~26%). There was a significant positive correlation between the content of leaf total sugars and the proportion of low-grade tobacco leaves (*r* = 0.72, *p* < 0.01). These findings underscore the vital influence of sugar-alkaloid balance and pyrolysis products on tobacco quality. Furthermore, K^+^ accumulated to higher levels in leaf samples, potentially because *S. rochei* D74 mediated K activation in soil, allowing efficient uptake of K^+^ ions during tobacco maturation. Our results are in line with previous findings of Zhu et al. (2024) regarding microbial regulation of soil fertility and plant nutrition.

Economic trait analysis revealed that the BA-FS treatment increased tobacco yield with a greater proportion of high-grade leaves. Ding et al. (2024) also observed that tobacco quality and economic benefits were improved after application of *Bacillus* inoculants. It has been reported that *S. fradiae* NKZ-259 benefits plant growth directly or indirectly by producing bioactive substances, such as phytohormones (e.g., indoleacetic acid), antibiotics, and vitamins (Myo et al., 2019). Although the present study did not directly quantify specific microbial metabolites (e.g., phytohormones, siderophores, or antibiotics), similar mechanistic pathways can be inferred for *S. rochei* D74 based on related literature. By extension, we hypothesize that *S. rochei* D74 may enhance tobacco root morphogenesis—as indicated by increased root length and surface area—potentially via phytohormone-mediated stimulation. This improved root architecture could subsequently facilitate the uptake of trace elements (e.g., Cu, Mo) and help coordinate leaf secondary metabolism. Taken together, these inferred mechanisms support a speculative “root–nutrient–metabolite” cascade regulation model, which remains to be directly validated in future studies.

### 4.3 *S. rochei* D74 mediates shifts in rhizosphere microbial community structure and pathogen abundance

Rhizosphere microbial community structure determines the health of soil ecosystems (Wu et al., 2016). This study revealed a reduction in fungal diversity within the rhizosphere soils of tobacco following microbial treatments, a shift primarily driven by a decline in *Fusarium* abundance, whereas bacterial diversity remained largely unaltered. This means that fungal communities responded more sensitively to *S. rochei* D74 than bacterial communities. Crowther et al. (2014) found that fungi are more susceptible to environmental disturbances, as they typically exhibit relatively slow growth rates and highly specific nutritional requirements. While a general loss of microbial diversity can compromise ecosystem resilience and functional redundancy, the context-dependent nature of this change is crucial. The observed reduction in fungal diversity may reflect a community restructuring process under the selective pressure exerted by *S. rochei* D74. It is plausible that this restructuring could preferentially affect certain pathogenic or competitive fungal taxa, potentially freeing ecological niches for beneficial microorganisms and thereby contributing to a more favorable resource allocation within the rhizosphere (Li et al., 2024). Taken together, these correlative findings are framed as associative evidence, linking plant growth promotion by *S. rochei* D74 with a restructured rhizosphere microbiome, which may thereby contribute to improved soil health and ecosystem stability.

Notably, the exceptionally high relative abundance of *Fusarium* in the control rhizosphere serves as a clear indicator of compromised soil health and a heightened risk of soil−borne disease. Under this substantial pathogen pressure, the BA-FS treatment effectively suppressed key fungal pathogens, notably reducing *Fusarium* abundance, a result consistent with the findings of Zhou et al. (2025). The marked improvements in soil quality achieved by *S. rochei* D74 demonstrate its ability to lower soil−borne disease risk. This inhibitory effect on soil-borne pathogens likely stems from antimicrobial metabolites produced by *S. rochei* D74 (e.g., *Streptomyces*-derived antimicrobial peptides) (Sun et al., 2024). Such antimicrobial mechanisms have also been reported in other *Streptomyces* species. For instance, Le et al. (2021) demonstrated that *Streptomyces* sp. JCK-6131 produces streptothricin derivatives capable of suppressing diverse plant pathogens. Similarly, Ahsan et al. (2017) reported that *Streptomyces* sp. KX852460 synthesizes eicosane and dibutyl phthalate, which exert a combined action against *Rhizoctonia solani* AG-3.

Mantel analysis revealed a significant correlation between *Fusarium* abundance in rhizosphere soils and K^+^ content in tobacco leaves. Secondary metabolites produced by *Fusarium* (e.g., fusaric acid, mycotoxins) may interfere with K^+^ uptake and transport in plants by disrupting mitochondrial function, inhibiting energy metabolism, and altering cell membrane permeability (Sardans and Peñuelas, 2021). Whether the direct interference of K^+^ uptake pathways by *Fusarium* mycotoxins affects plant health and disease resistance in tobacco still requires validation. Despite this, our study provides crucial evidence that *S. rochei* D74 plays a role in controlling soil-borne pathogens and maintaining plant health in tobacco fields.

## 5 Conclusions

This study demonstrated the effects of *S. rochei* D74 on tobacco growth and quality from multiple dimensions—microbial ecology, soil chemistry, and plant physiology. *S. rochei* D74 mediated the additive enhancement of tobacco growth and quality by restructuring of rhizosphere microbial communities, activation of soil nutrient cycling, and coordination of leaf chemical composition. The combinatorial effect primarily stemmed from the suppression of soil−borne pathogens, particularly reflected in the reduced relative abundance of *Fusarium*, which is closely associated with improved soil health and lowered disease risk. Taking into account soil properties and tobacco traits, basal application combined with spray foliar was the optimal method for field application of *S. rochei* D74. The findings provide reliable technical support for mitigating soil degradation and enhancing tobacco yield and quality in continuous cropping systems.

## Data Availability

The raw sequencing data for bacterial 16s rRNA gene and fungal ITS regions have been deposited in NCBI SRA database using accession code PRJNA1398861.
