# Supplementary material for: Streptomyces rochei D74 improves tobacco growth and quality by regulating the rhizosphere microecological community
Source: Front Plant Sci. 2026 Mar 5;17:1748408. doi: 10.3389/fpls.2026.1748408 (PMC12999567; doi:10.3389/fpls.2026.1748408)
Supplement: Supplementary file 1 [file DataSheet1.docx]

Supplementary Material

# Soil Physicochemical Properties of the Study Area

**Supplementary Table 1.** Basic physicochemical properties of the soil.

| **Soil depth**  **(cm)** | **pH value** | **Organic matter**  **(g/kg)** | **Hydrolyzable N**  **(mg/kg)** | **Available P**  **(mg/kg)** | **Available K**  **(mg/kg)** | **Total N**  **(g/kg)** | **Available Zn**  **(mg/kg)** |
| --- | --- | --- | --- | --- | --- | --- | --- |
|  |  |  |  |  |  |  |  |
| 0–20 | 7.75 | 15.41 | 77.70 | 7.80 | 160.03 | 0.67 | 0.35 |
| **Soil depth**  **(cm)** | **Available Mn**  **(mg/kg)** | **Available Fe**  **(mg/kg)** | **Available Cu**  **(mg/kg)** | **Available B**  **(mg/kg)** | **Exchangeable Ca**  **(mg/kg)** | **Exchangeable Mg**  **(mg/kg)** | **Cl^-^**  **(mg/kg)** |
|  |  |  |  |  |  |  |  |
| 0–20 | 7.00 | 2.30 | 0.33 | 0.15 | 4397.02 | 36.33 | 7.20 |

#
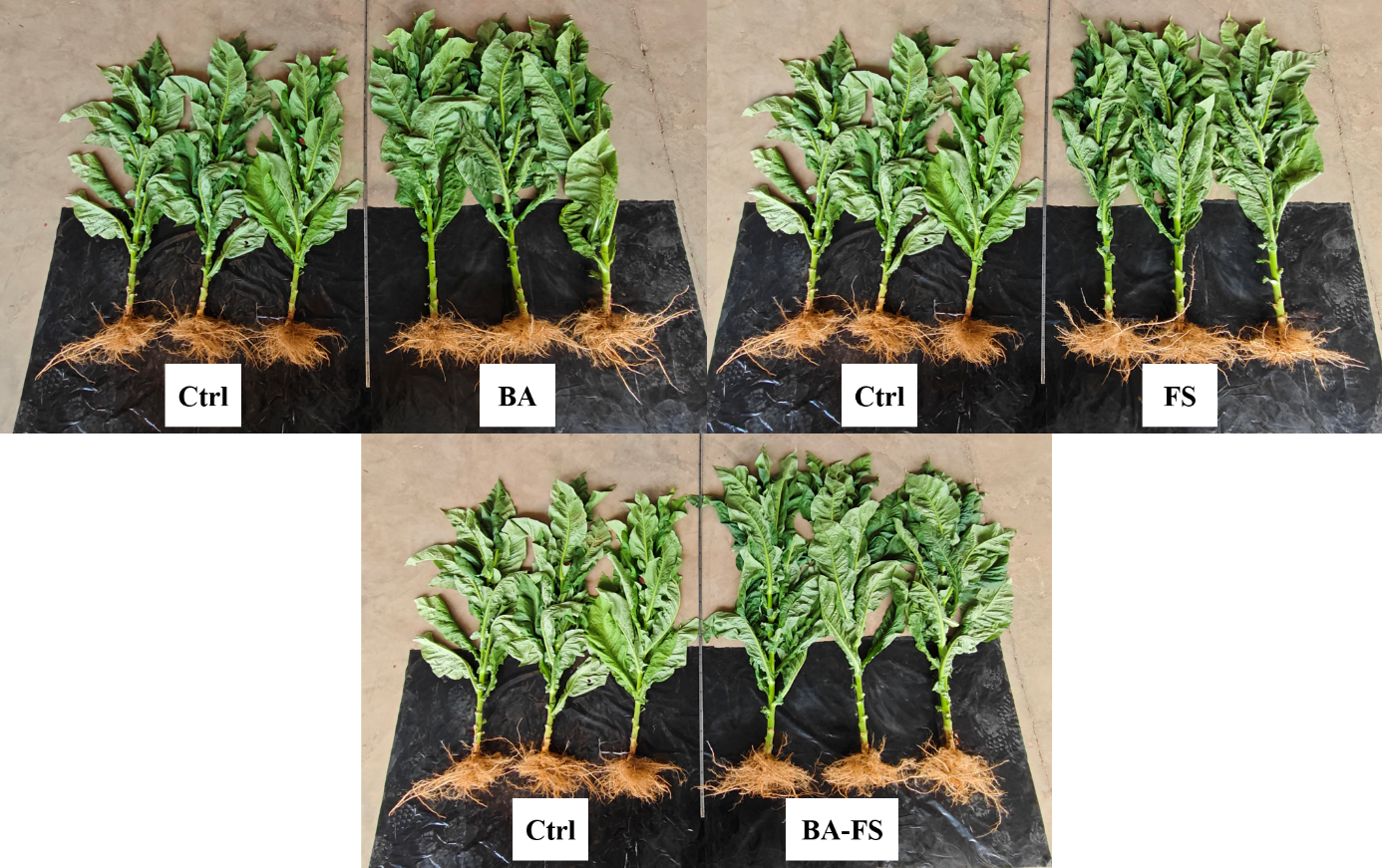
Photographs of Plant Phenotypes

**Supplementary Figure 1.** Plant and root morphological characteristics of flue-cured tobacco in response to different application strategies of *S. rochei* D74. Ctrl = Sterile organic fertilizer (root) + sterile medium (foliar spray); BA = basal application; FS = foliar spray; BA-FS = basal application combined with foliar spray.

# Membership Function Model Method


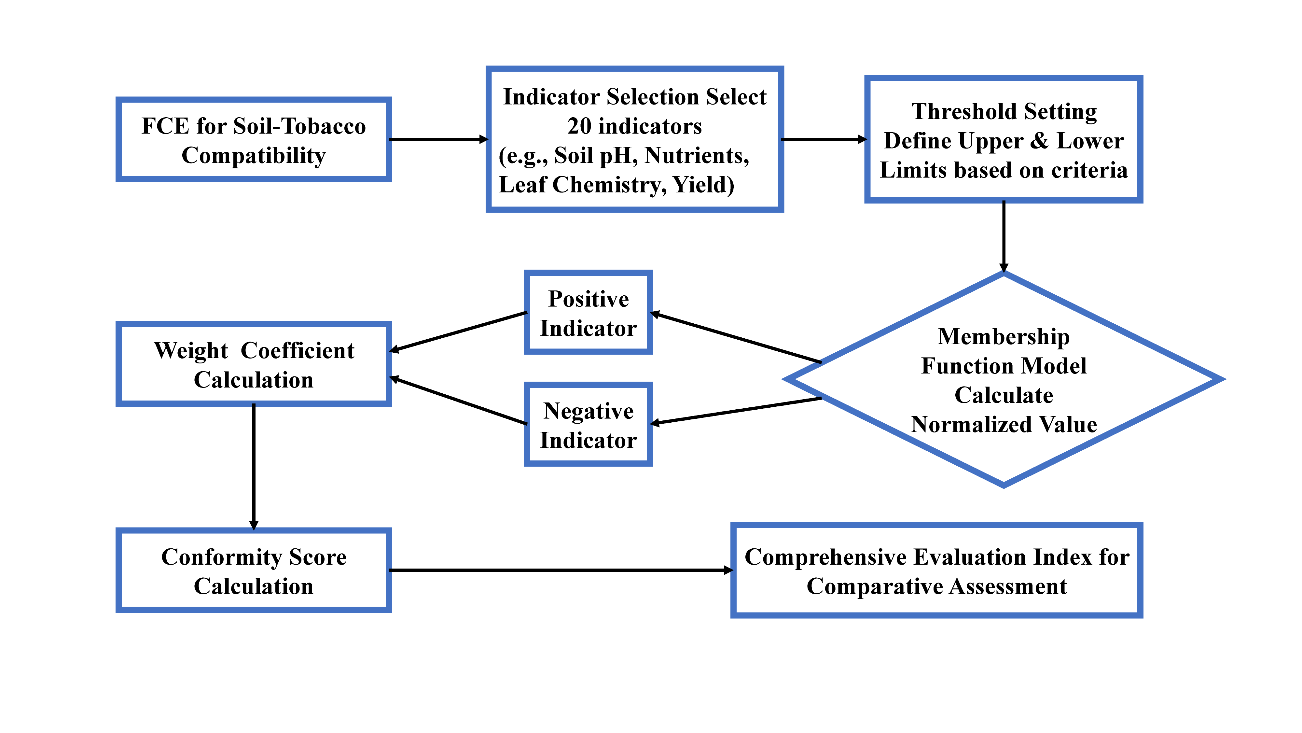
The membership function model was employed to normalize soil nutrients and conventional chemical composition indices of tobacco leaves into dimensionless values ranging from 0 to 1. Subsequently, a fuzzy comprehensive evaluation (FCE) method was applied to compute a comprehensive evaluation index, which reflects the compatibility between soil physicochemical properties and tobacco leaf yield and quality. This index was further quantified to determine its contribution, thereby enabling a comparative assessment of soil fertility and tobacco quality across the different treatments. The schematic diagram is illustrated as follows (Supplementary Figure 2).

**Supplementary Figure 2.** Fuzzy Comprehensive Evaluation (FCE) Flowchart

## Indicator Selection

Each application method selected 20 indicators such as soil pH, nutrients, trace elements, chemical composition and yield of tobacco leaves.

## Threshold Setting

The upper and lower limits for each indicator in the membership function model were defined based on the established criteria for soil nutrients and conventional chemical composition of high-quality tobacco leaves (Supplementary Table 2).

**Supplementary Table 2.** The upper and lower limits of soil and plant indicators

| Index | Lower limit (L_j_) | Upper limit (U_j_) | Direction (D_j_) |
| --- | --- | --- | --- |
| Soil pH value | 7.7 | 7.8 | positive |
| Soil organic matter | 15 | 20 | positive |
| Soil hydrolyzable N | 60 | 120 | positive |
| Soil available P | 18 | 25 | positive |
| Soil available K | 400 | 500 | positive |
| Soil available Zn | 0.5 | 1.5 | positive |
| Soil available Mn | 5 | 10 | positive |
| Soil available Fe | 4.5 | 10 | positive |
| Soil available Cu | 0.5 | 2 | positive |
| Soil exchangeable Ca | 2000 | 3000 | positive |
| Soil Cl^-^ | 1 | 10 | negative |
| Leaf total N | 1.8 | 2.4 | positive |
| Leaf total alkaloids | 1.5 | 3.5 | positive |
| Leaf total sugar | 28 | 32 | positive |
| Leaf reducing sugar | 18 | 24 | positive |
| Leaf K^+^ | 4 | 10 | positive |
| Leaf Cl^-^ | 0.1 | 0.6 | positive |
| Leaf K/Cl | 4 | 10 | positive |
| Leaf starch | 0 | 5 | positive |
| Production | 148.52 | 160.02 | positive |

Note: A positive direction denotes that larger values are more desirable, and a negative direction denotes that smaller values are more desirable.

## Membership Function Model

Ⅰ. Positive indicators

$\mu_{ij}=\left\{ \begin{aligned} 0.1 \\ 0.9\frac{x_{ij}-L_{j}}{U_{j}-L_{j}}+0.1 \\ 1 \end{aligned} \right.\begin{matrix} x_{ij}\leq L_{j}; \\ L_{j}<x_{ij}<U_{j}; \\ x_{ij}\geq U_{j}. \end{matrix}$ (1)

Ⅱ. Negative indicators

$\mu_{ij}=\left\{ \begin{aligned} 1 \\ \frac{{U_{j}-x}_{ij}}{U_{j}-L_{j}} \\ 0 \end{aligned} \right.\begin{matrix} x_{ij}\leq L_{j}; \\ L_{j}<x_{ij}<U_{j}; \\ x_{ij}\geq U_{j}. \end{matrix}$ (2)

Note: $i$: Sample index, denoting the i-th evaluation object; $j$: Indicator index, denoting the j-th evaluation indicator; $x_{ij}$: Original measured value of the j-th indicator for the i-th sample; $L_{j}$: Lower threshold for the j-th indicator. When $x_{ij}\leq L_{j}$, it signifies a relatively poor performance regarding this indicator; $U_{j}$: Upper threshold for the j-th indicator. When $x_{ij}\geq U_{j}$, it signifies a favorable performance regarding this indicator; $\mu_{ij}$: Membership degree of the j-th indicator for the i-th sample. It quantifies the degree to which the indicator belongs to the "favorable" category, typically ranging from 0 to 1.

## Weight Coefficient Calculation

Using the average absolute correlation coefficient method:

Ⅰ. Calculate the Pearson correlation matrix

$R={(r_{jk})}_{m\times m}$ (3)

Ⅱ. Compute the average absolute correlation coefficient for each indicator

$\bar{r}_{j}=\frac{1}{m}\sum_{k=1}^{m} |r_{jk}|$ (4)

Ⅲ. Normalization to determine weight coefficients

$w_{j}=\frac{\bar{r}_{j}}{\sum_{j=1}^{m} \bar{r}_{j}}$ (5)

$\sum_{j=1}^{m} w_{j}=1$ (6)

Note: $R$: Pearson correlation matrix, a symmetric matrix of dimension $m\times m$; $r_{jk}$: Pearson correlation coefficient between the k-th indicator and the j-th indicator. It measures the linear correlation between two indicators, with values ranging from -1 to 1; $m$: Total number of evaluation indicators; $\bar{r}_{j}$: Average absolute correlation coefficient for the j-th indicator; $w_{j}$: Weight coefficient assigned to the j-th indicator, $0\leq w_{j}\leq1$.

The weight coefficients of soil and plant indicators are summarized in the following Supplementary Table 3.

**Supplementary Table 3.** Weight coefficient of soil and plant indicators

| Index | Weight | Ranking number |
| --- | --- | --- |
| Soil pH value | 0.051950938 | 9 |
| Soil organic matter | 0.051592188 | 10 |
| Soil hydrolyzable N | 0.052310927 | 8 |
| Soil available P | 0.052844034 | 7 |
| Soil available K | 0.050912698 | 12 |
| Soil available Zn | 0.053299843 | 6 |
| Soil available Mn | 0.055036105 | 4 |
| Soil available Fe | 0.055306912 | 1 |
| Soil available Cu | 0.035997136 | 20 |
| Soil exchangeable Ca | 0.055179444 | 2 |
| Soil Cl^-^ | 0.05512403 | 3 |
| Leaf total N | 0.050250083 | 14 |
| Leaf total alkaloids | 0.037318389 | 19 |
| Leaf total sugar | 0.05121192 | 11 |
| Leaf reducing sugar | 0.049511788 | 15 |
| Leaf K^+^ | 0.050499143 | 13 |
| Leaf Cl^-^ | 0.048013481 | 16 |
| Leaf K/Cl | 0.05458432 | 5 |
| Leaf starch | 0.045107882 | 17 |
| Production | 0.04394874 | 18 |

## Conformity Score Calculation

The conformity score of soil physicochemical properties and tobacco yield and quality

$\sum_{j=1}^{m} w_{j}\mu_{ij}$ (7)

#
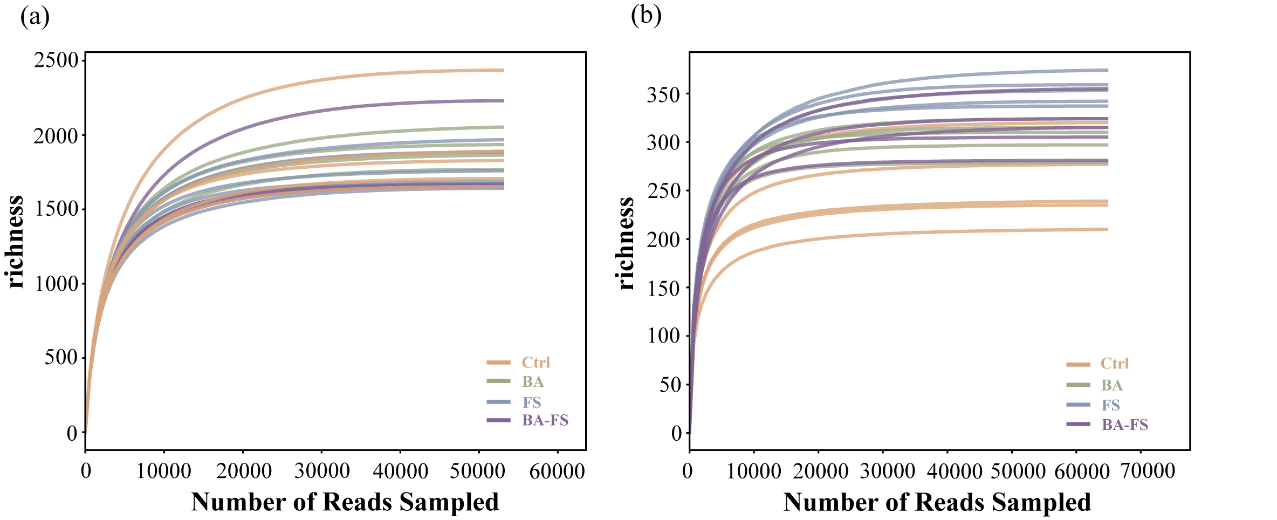
Standardized Sampling Analysis of Microbial Community Sequencing Data

**Supplementary Figure 3.** Rarefaction curves of rhizosphere microbiota across treatments. (a) Bacterial community; (b) Fungal community.

# Treatment Effects on the Chemical Quality of High-Grade Tobacco Leaves

**
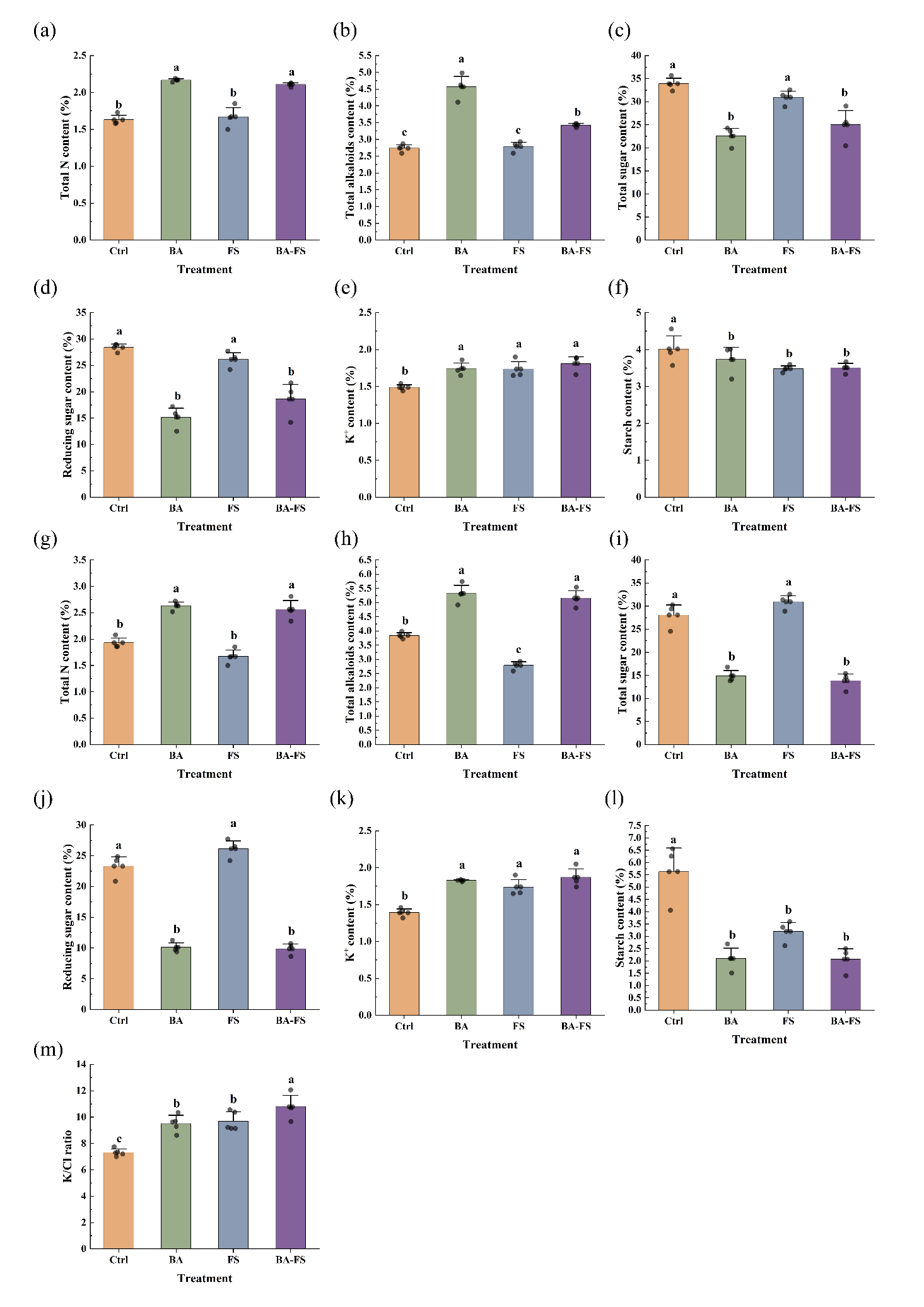
**

**Supplementary Figure 4.** Changes in chemical quality of C3F (a–f) and B2F (g–l) grade tobacco leaves with different microbial application methods. (a, g) Total N content. (b, h) Total alkaloids content. (c, i) Total sugar content. (d, j) Reducing sugar content. (e, k) K^+^ content. (f, l) Starch content. (m) K/Cl ratio in C3F and B2F grade leaves. Ctrl = Sterile organic fertilizer (root) + sterile medium (foliar spray); BA = basal application; FS = foliar spray; BA-FS = basal application combined with foliar spray. Error bars represent standard deviation of the mean (*n* = 5). Different letters above the error bars indicate significant differences among treatments (*p* < 0.05).

#
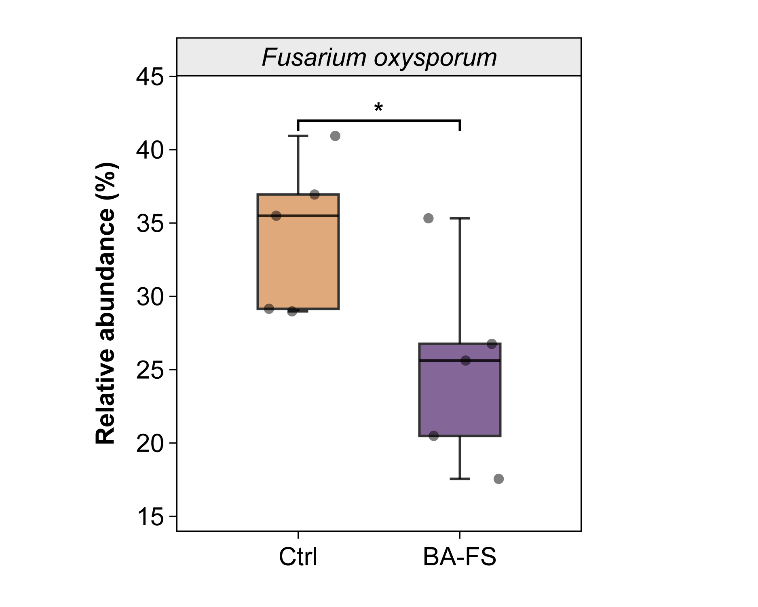
Effects of Treatments on Rhizosphere Microbiota


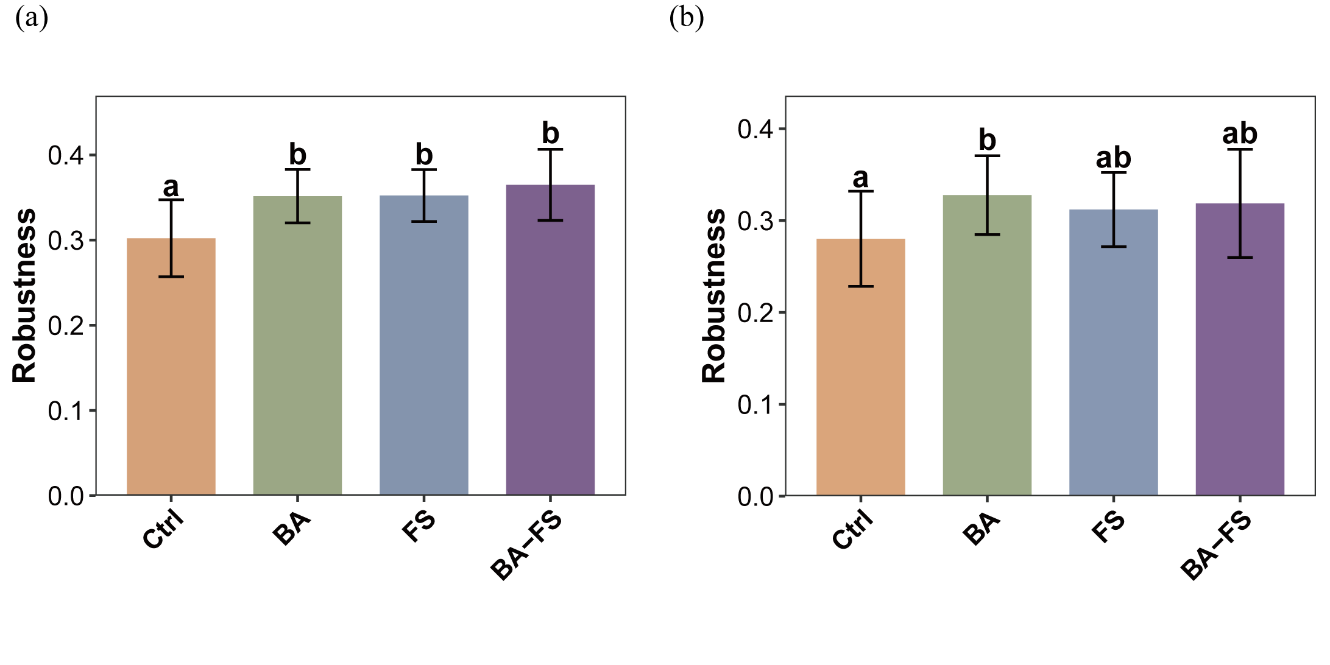
**Supplementary Figure 5.** Relative abundance of *Fusarium oxysporum*. Error bars represent standard deviation of the mean (*n* = 5). Significant differences between treatment means are indicated by asterisks above the error bars (*p* < 0.05).

**Supplementary Figure 6.** Stability evaluation of bacterial and fungal networks. (a) Bacterial network robustness; (b) Fungal network robustness. Ctrl = Sterile organic fertilizer (root) + sterile medium (foliar spray); BA = basal application; FS = foliar spray; BA-FS = basal application combined with foliar spray. Error bars represent standard deviation of the mean (*n* = 5). Different letters above the error bars indicate significant differences among treatments (*p* < 0.05).
